# Supplementary material for: Are carbon emissions trading and green financial instruments synergistic? -Comprehensive quantitative research based on content analysis
Source: PLoS One. 2024 Mar 7;19(3):e0298601. doi: 10.1371/journal.pone.0298601 (PMC10919601; doi:10.1371/journal.pone.0298601)
Supplement: S1 Data — (ZIP) [file pone.0298601.s001.zip › Data/地级市Z_F工作报告.zip!/║╙▒▒/▓╫╓▌╩╨/└┤╫╘╣1⁄2╓┌║┼í╛┬φ┐╦╩2╛▌═°í┐_2014.docx]

各位代表：

现在，我代表沧州市人民政府，向大会作工作报告，请予审议，并请各位政协委员和其他列席会议的同志提出意见。

一、2013年工作回顾

刚刚过去的一年，是我市改革发展进程中不寻常、不平凡的一年，也是全市上下抢抓机遇、开拓进取，各项工作取得显著成效的一年。全市人民在省委、省政府和中共沧州市委的正确领导下，深入贯彻落实党的十八大和十八届三中全会精神，紧紧围绕打造河北沿海地区率先发展增长极目标，统筹推进“三大经济板块”，培育壮大“七大增长点”，着力调结构、稳增长、优环境、惠民生，较好完成了市十三届人大一次会议确定的各项任务目标。全市地区生产总值突破3000亿元，预计完成3020亿元，增长9%；全部财政收入突破400亿元，达到416.5亿元，公共预算收入172.3亿元，可比分别增长11.2%和23.4%；固定资产投资2360亿元，增长21.1%；规模以上工业增加值1271.8亿元，增长11.5%；城镇居民人均可支配收入和农民人均纯收入分别达到22885元和8415元，增长10%和12%。

（一）“三大经济板块”协调发展，综合经济实力快速提升

沿海开发建设实现新突破。黄骅港综合港区二期工程取得重大进展，20万吨级航道和矿石码头建成试运行，邯黄铁路通车到渤海新区，向现代化综合大港目标迈出了坚实一步。全年完成吞吐量1.7亿吨，增长35.4%，增速居全国主要港口首位。集装箱23万标箱，实现翻番增长。中捷石材城、新加坡金鹰集团LNG码头等重点项目签约落地。中国一重冷轧薄板、中海油中捷石化炼化一体化、渤海新区2×35万千瓦热电机组等137个亿元以上在建项目顺利推进。北汽40万辆整车一期、新启元30万吨蒽油加氢等一批项目竣工投产。出台《关于扶持物流贸易发展的优惠政策》，新增物流贸易企业486家，华北大宗商品交易中心上线运营。沧州临港经济技术开发区获“国家新型工业化产业示范基地”称号。

中心城区经济呈现新气象。沧州开发区、高新区、沧东开发区和新华、运河工业园投入力度不断加大，全年完成固定资产投资106.5亿元，增长41%。航天神舟太阳能光热产业园、中国机械机器人产业园、中科招商产业园、台商产业转移创新基地等一批高新技术和先进制造业项目扎实推进。明珠商贸城、国际五金城、义乌小商品城、佳兴仓储物流开工建设。泰大国际家居博览中心、颐和新世界、彩龙国际商贸广场建成运营。沧东物流园区晋升为省级物流产业聚集区。

县域经济发展跃上新台阶。特色产业快速发展，聚集效应日益凸显。销售收入超百亿元产业集群达到7个，超50亿元20个。新增产业集群龙头示范企业32家，总数达到41家，居全省第一。盐山县荣获“中国管道管件出口基地”称号。吴桥中汽轮胎、河间华旗线缆等161个亿元以上项目开工建设，河北一塑聚乙烯管材、肃宁裘皮城二期等91个项目竣工投产。所有县（市）全部财政收入超4亿元，任丘市超百亿元。

（二）“七大增长点”增势强劲，支撑带动作用明显增强

重点项目建设步伐加快。推进实施重点项目领导分包和“4+2”观摩考评机制，全市亿元以上竣工和在建项目536个，总投资4292亿元，累计完成投资1207亿元。其中，新开工253个，竣工投产130个，新竣工入统规模以上工业企业31家。实施投资超100亿元项目8个，百万吨乙烯、80万吨PX、海兴核电等一批战略支撑项目取得突破性进展。沧州项目多、块头大、质量好、后劲足，推进重点项目建设典型做法在全省经济工作会议上专题播放。

工业经济实力不断壮大。以技术改造和科技创新为手段，加快转型升级步伐。科林机械、沧海重工等137个亿元以上技改项目扎实推进，完成投资189亿元。龙头骨干企业支撑带动作用明显增强，中石油华北石化、中铁装备新材料等50强工业企业，完成主营业务收入和税收分别占全市规模以上工业的42%和80%。企业“登台阶”计划稳步实施，全年新增规模以上工业企业258家，其中，规下升规上227家。新增国家高新技术企业26家，高新技术产业完成增加值200亿元，增长20%。

城乡面貌发生显著变化。中心城区“十大城建工程”顺利推进，沧州体育场、图书馆建成投用，博物馆、游泳馆开馆在即，管业大厦、传媒大厦等5个企业总部主体完工。广州路、海丰大道等9条城市道路竣工通车。宏宇城、皇家壹里等住宅小区交工入住，新增商品住房面积94.8万平方米。东部城区集中供热工程投入运行，新增供热面积61万平方米，17个住宅小区完成二次供热管网改造。绿色行动三年计划成效显著，滨河公园、体育公园等4个公园和10个街头游园建成开放，新增绿化面积263.4公顷，建成区绿化覆盖率达到41.5%。全市完成植树造林21.2万亩，森林覆盖率24.5%。县城建设全面推进，33个精品工程完成投资30.8亿元，沧州市区、河间、黄骅获河北省人居环境进步奖。448个省级重点村完成面貌改造提升任务。

服务业水平进一步提升。传统服务业扩规提质，现代服务业加快发展，全年完成增加值1094.2亿元，增长8.2%。新增限额以上企业69家，总数达到541家，实现新增零售额13亿元。全市拥有亿元以上大型商品交易市场40个，年交易额458.7亿元。消费市场持续繁荣，完成社会消费品零售总额886.2亿元，增长13.7%，电子商务、网络购物成为消费新热点。

园区开发建设力度加大。19个省级以上开发区和工业聚集区，完成固定资产投资1114.9亿元、主营业务收入3653.3亿元、税收187.3亿元，分别增长26.2%、19.5%和17.7%。任丘开发区主营业务收入超800亿元，沧州开发区、河间市新区等5个园区超200亿元。省政府批复设立省级中捷高新技术产业开发区。

农业生产持续快速发展。粮食总产达到477.4万吨，我市被农业部评为“全国粮食生产先进市”。渤海粮仓科技示范工程取得重要成果，示范推广面积达到3万亩。新增市级以上农业产业化龙头企业57家，总数达到503家。新增农民专业合作组织2507家，总数达到4995家。新建改造10千伏线路904公里、配变站2340台、农村公路1300公里，铺设地下输水管道3850公里，发展节水灌溉面积45万亩。

改革开放积极有序推进。制定实施《进一步支持全民创业的十条措施》、《改革企业登记制度实施办法》等系列支持政策，全年新增各类市场主体2.86万户，是上年新增数量的3.5倍。加快推进行政审批制度改革，全市取消、下放行政审批事项73项，审批时限和审批环节分别精简65.1%和22.7%。成功举办沧州—西安经济技术合作恳谈会、冀台经济合作、沧州投资环境说明暨侨商合作洽谈会。全年实际利用外资4.1亿美元，增长15.2%；引进省外资金630亿元，增长25%；完成外贸进出口25.2亿美元，增长8%。积极引进域外股份制银行，加快企业上市步伐，新增上市企业20家，总数达到52家，居全省第一位。

（三）民生工程扎实推进，社会各项事业全面进步

加大民生建设投入，全年财政用于民生支出257亿元，占公共财政预算支出的75%。“十大民生工程”顺利实施，开工建设保障性住房2.97万套，2.8万户居民乔迁新居。新增城镇就业5.65万人，转移农村劳动力5.3万人，城镇登记失业率控制在3.8%以内。企业退休人员养老金待遇人均月增178元，城乡居民养老保险参保率达到94.6%。城镇居民医疗保险和新农合财政补助标准人均提高到280元，城乡低保标准分别提高到4464元和2243元。南水北调配套工程开工建设，完成投资8.5亿元。投入扶贫开发资金25.1亿元，8万人实现稳定脱贫。改造农村危房2.4万套，87万人饮水安全问题得到解决。生态环境保护和大气污染防治工作取得阶段性成果，主要污染物减排任务基本完成。淘汰“黄标车”8.7万辆，更新LNG公交车191部、客运车150部。教育、文化、体育、卫生事业稳步发展，创建标准化中小学50所，改造农村薄弱学校47个，全部消除D级危房，3万余名进城务工人员子女义务教育得到保障。成功举办第十四届中国吴桥国际杂技艺术节沧州会场。新建改建28所乡镇卫生院和3987个集体产权村卫生室，10家县级改革试点医院全部实行药品零差率销售。食品药品安全监管力度不断加大，安全生产形势持续好转。平安沧州建设扎实推进，社会大局和谐稳定。

（四）行政效能大幅提高，政府自身建设不断加强

组织开展对标先进活动，创新方式方法，提速行政效能，服务经济发展。政务服务中心、公共资源交易中心，以及企业“110”和金融服务中心实现规范运转，42家单位436个事项实现集中审批、公开办理。严格执行中央“八项规定”，认真开展正风肃纪专项行动，查处违规违纪行为166件，对253名涉事人员给予严肃处理。全面清理公务用车、超标办公用房，市直机关“三公”经费支出压减7%左右。主动向人大报告工作、向政协通报情况，全年共办理人大代表建议135件，政协提案341件，按时办复率100%。

各位代表，奋斗历尽艰辛，成绩来之不易。一年来，全市上下面对风险和挑战，奋力拼搏，扎实苦干，经济发展稳中向好、稳中有进，多项指标和增速在全省保持较好名次；城市建设日新月异，竞争力、影响力快速提升，在福布斯中文版发布的中国大陆最佳商业城市排行榜中，我市首次跻身百强，在全国650个建制城市中名列第95位；城乡居民生活条件持续改善，社会安定和谐有序，人民群众的幸福感、安全感有了新的提高。这些成绩的取得，是中共沧州市委正确领导的结果，是市人大、市政协关心支持的结果，是全市各级干部共同努力奋斗的结果，是全市人民团结一心、攻坚克难的结果。在此，我代表沧州市人民政府，向辛勤工作在各条战线上的全市各族人民，向给予我们大力支持的人大代表、政协委员和各民主党派、工商联、无党派人士，向离退休老干部、驻沧各单位、人民解放军和武警官兵，向所有关心、支持沧州发展的同志们、朋友们，致以崇高的敬意和衷心的感谢！

在总结成绩的同时，我们也清醒地认识到，发展中仍存在一些亟待解决的矛盾和问题，主要是：经济总量还不够大，发展水平还不够高；产业升级、结构调整任务重，现代服务业和战略性新兴产业发展相对滞后；对外开放水平低，与沿海区位不相称；政府职能转变任务艰巨，一些部门和工作人员服务意识淡薄、作风简单粗暴，“吃拿卡要”现象还不同程度地存在。对这些问题，我们一定高度关注，认真对待，采取更加积极的措施，下大力加以解决，决不辜负全市人民对我们的信任和期待！

二、2014年发展目标和总体要求

今年是全面贯彻落实党的十八大、十八届三中全会精神的重要一年，也是打造河北沿海地区率先发展增长极、全面深化改革、加速转型升级的关键一年。当前，我市发展面临的外部环境和内部条件都在发生深刻复杂的变化，世界经济复苏缓慢，国内资源能源和环境约束压力加大，产业转型升级任务艰巨。但从发展总体趋势看，我国仍处于重要战略机遇期，新型工业化、新型城镇化步伐加快，内生需求持续增加。党的十八届三中全会拉开了全面深化改革的序幕，将进一步激发我国经济增长的动力和活力。随着《河北沿海地区发展规划》深入实施、首都经济圈和环渤海经济圈一体化快速推进，我省在京津冀协同发展中的战略地位日益突出，发展机遇进一步显现。经过长期不懈努力，我市已具备跨越赶超的优势和条件，拥有率先发展的基础和实力。我们一定要树立机遇意识、进取意识，切实增强加快发展的责任感、紧迫感，积极作为、善作善为，以时不我待、奋勇争先的决心和勇气，努力开创沿海强市建设新局面！

今年政府工作的总体要求是：全面贯彻落实党的十八大、十八届三中全会、省委八届六次和市委八届四次全会精神，坚持稳中求进、变中求快工作总基调，围绕打造河北沿海地区率先发展增长极目标，突出转变、增长、改革、开放、项目、实干，继续坚定不移地实施沿海开放带动战略，做大做强“三大经济板块”，抓实“七大经济增长点”，促进经济健康快速发展，人民生活水平稳步提高，努力建设靓丽、繁华、宜居、和谐新沧州。

主要预期目标是：全市地区生产总值增长9%，公共财政预算收入增长10%，全社会固定资产投资增长19%，规模以上工业增加值增长12%，城镇居民人均可支配收入和农民人均纯收入分别增长10%，居民消费价格指数涨幅控制在3.5%以内，人口自然增长率控制在8.7‰以内，单位生产总值能耗和主要污染物排放达到省下达的控制目标。需要说明的是，虽然主要指标的设定比上年略有下调，但均高于全省平均增长水平，体现了既积极又稳妥的原则和打造增长极的要求。确定地区生产总值增长9%，不再设置全部财政收入指标，强调节能减排等约束性指标，主要目的是按照省委、省政府“绿色崛起”要求，转变发展的指导思想，不以GDP增长论英雄，努力实现更高质量、更高水平的发展。

完成上述目标，需要付出艰苦的努力。工作中我们将注意把握以下几个方面：一是坚持把“转变”作为工作主线。切实转变片面追求发展速度、过度依赖资源能源消耗的思想意识和发展方式，更加注重产业转型升级，更加注重科技创新，更加注重循环发展、低碳发展，在转变中实现绿色崛起。二是坚持把“增长”作为首要任务。着眼巩固平稳较快增长好局面，努力克服不稳定、不确定因素影响，稳中有为、稳中有进，坚定不移加快发展，确保经济实现适度较快增长。三是坚持把“改革”作为核心动力。将改革创新贯穿于经济社会发展各领域、各环节，以更大的勇气和智慧推进重点领域改革，以改革解放和发展生产力、解放和增强社会活力。四是坚持把“开放”作为重要手段。牢固树立沿海意识、环京津意识，充分运用开放的思维和开放的办法，统筹国际国内两个市场、两种资源，加快引进央企、外企和大型民企，不断提高对外开放层次和水平。五是坚持把“项目”作为重要支撑。牢固树立“项目是打造河北沿海地区率先发展增长极最重要支撑”的理念，坚持一产、二产、三产项目一起抓，大、中、小项目一起上，以项目调结构，以项目夯基础，以项目增后劲。六是坚持把“实干”作为根本要求。按照“四个干”要求，把各项决策部署落实到事，落实到人，落实到具体措施，落实到时间节点，跟踪督导，加压问责，务求实效。

各位代表，发展的目标激励着我们，严峻的形势考验着我们，肩负的责任鞭策着我们。只要我们咬定青山不放松、面对困难不退缩、团结奋斗不懈怠，就一定能够实现全年任务目标，在建设沿海强市、和谐沧州的征程中迈出更加坚实有力的步伐！

三、2014年主要任务

重点抓好八个方面的工作

（一）统筹发展“三大经济板块”。坚持着眼全局，统筹兼顾，加快推进沿海经济、中心城区和县域经济协调发展，形成各具特色、支撑有力、优势互补的发展格局。

全力推进沿海经济。充分发挥渤海新区率先发展的引领作用，带动沿海经济扩规模、上水平。抓好20万吨级航道和20万吨级矿石码头运营和配套设施建设，加快推进总投资374亿元的32个专业码头，力争新开工各类码头8个以上。积极引进大型航运公司、船舶公司，开辟国内外航线；加快完善查验、联检设施和通关能力建设，确保黄骅港口岸正式开放，力争全年完成吞吐量2.3亿吨，集装箱35万标箱。组织实施“千亿产业园区计划”，加快打造黄骅汽车产业园、中捷石化产业园、临港石化产业园等一批销售收入超千亿元园区。积极推进神华机车检修中心、正元化肥合成氨及尿素等一批亿元以上重点项目，力争69个项目竣工投产。做大做强临港物流贸易，加快建设煤炭、矿石、建材、化学品等“八大交易中心”和电子商务、综合保税“两大服务平台”，打造临港物流集散基地，实现物流贸易交易额翻一番。力争渤海新区综合保税区获国家批准。推动石港、邯黄高速公路及石沧港客货铁路前期工作，加快供水、供电、供热、供气等配套设施建设。面向朔黄、邯黄铁路沿线，着眼构筑“1+4”格局，推进“内陆港”建设，实施“海铁路联运”，带动冀中南等腹地区域加快发展。加快黄骅新城建设，提升临港商务区功能，着力打造生态宜居滨海新城。

发展壮大中心城区经济。加大政策扶持和协调推进力度，支持“四区一县”大力发展高新技术产业和现代服务业，加快建设繁华沧州。统筹“三区两园”发展，进一步优化空间布局，完善基础设施，提升承载能力。推进实施一批科技含量高、市场前景好的新型工业化项目，重点抓好中国机械机器人产业园、中科招商产业园、沧州国际电子商务产业园、森茂特激光产业园、台商产业转移创新基地等重点工程。加快千童商业广场、华信物流配送中心等15个商贸项目建设，确保荣盛锦绣天地、明珠商贸城、沃尔玛购物中心建成运营，打造布局合理、高端现代的中心商务功能区和特色商业区。大力发展总部经济、金融证券、文化传媒、信息咨询等新兴服务业，不断提高服务业对中心城区经济的贡献率。

做大做强县域经济。创新工业园区管理机制，突出抓好项目建设和招商引资，打造产业转型升级平台，力争主营业务收入超200亿园区达到7个，所有园区主要经济指标增长25%以上。突出重点，分类指导，加快发展任丘石化产业，抓好华北石化千万吨炼油、百万吨乙烯、80万吨PX、PTA等一批石化产业项目，延伸链条，扩大产能，打造销售收入超千亿元的石化基地。支持盐山、孟村管道装备制造业上装备、上技术、上水平，加快建设国内外知名的“管道之都”,争取纳入省支持的用钢产业园区。鼓励泊头机床制造、南皮五金机电、东光纸箱机械等特色产业转型升级，与国内外大型装备企业实施战略重组、嫁接改造，实现由零件向部件，由部件向整机转变。全面落实支持中小微企业、促进民营经济发展的政策措施，支持民营企业通过科技创新、技术改造向规模企业、行业领军企业快速迈进。

（二）全力以赴抓好重点项目建设。坚持谋划储备一批、前期跑办一批、开工建设一批、竣工投产一批、入统达效一批，梯次推进重点项目建设，确保全年完成投资1400亿元。

抓战略支撑。紧紧抓住事关长远发展的38个战略支撑项目，明确时间节点，强化跟踪调度，确保顺利推进。突出抓好中石油华北石化、中海油中捷石化、中国一重300万吨冷轧薄板、旭阳化工40万吨己内酰胺、中捷石材城、中核集团海兴核电等17个投资超百亿元项目，千方百计抓落地、促开工、保投产，为经济增长聚集新能量。

抓省、市重点。加强谋划和跑办，储备重点项目1000个以上，力争更多项目进入国家和省建设计划。加大推进力度，确保河北华晨海洋医药、朔黄铁路联运基地等200个亿元以上重点项目开工建设，海特伟业碳四综合利用、河间瀛洲化工等100个项目竣工投产。加快实施石津、保沧干渠南水北调配套项目、京沪高速沧州段等一批建设工程，增强基础设施对经济发展的支撑能力。

抓项目管理。强化土地、资金等要素保障，引导优质资源向重点项目、产业支撑项目集中。落实重点项目领导分包责任制，健全“4+2”观摩考评机制，进一步调动全市争项目、跑项目、上项目的积极性、主动性。

（三）大力调整优化产业结构。坚持把产业结构调整放在更加重要位置，通过调优存量，扩大增量，提高质量，促进一产总量稳步扩大、二产结构不断优化、三产水平持续提升。

全面提升工业经济发展质量。加快工业强市建设步伐，深入实施“50强培育工程”，重点抓好宏润重工、华油钢管等50家企业，培育一批有规模、上档次、带动力强的行业龙头骨干，力争全年新增销售收入超百亿元企业1家，达到8家；10—100亿元7家，达到72家。继续抓好“1+1”技改工程，加快渤海重工、恒盛舰船专用泵等146个亿元以上技改项目建设，确保全年工业技改投资增长20%以上。坚持培育与引进并重、自主研发与市场开拓相结合，重点培育发展新材料、新能源、电子信息、生物医药等战略性新兴产业。加强工业运行监测分析，建立与重点企业联系制度，健全银企对接、产需衔接机制，解决好企业发展中的突出问题。

积极拓展服务业发展领域。完善落实支持政策，培育壮大“服务业50强”，推进服务业发展提速、比重提高、水平提升。积极发展生产性服务业，以现代物流业为重点，以渤海新区、中心城区和肃宁三大物流聚集区为平台，加快引进国际国内大型物流企业，大力发展大宗商品、电商交易，推进多式联运，构建集仓储、运输、信息服务于一体的综合物流系统，打造以黄骅港为龙头、联通海内外的华北地区大型物流集散中心。加快发展生活性服务业，推动商贸流通等传统服务业转型升级，培育发展文化娱乐、体育健身、旅游休闲、社区服务等新兴服务业。支持住房改善性需求，促进房地产市场健康发展。

大力发展现代高效农业。加快粮食生产核心区建设，稳定种植面积1300万亩。扎实推进“渤海粮仓科技示范工程”，完成示范推广面积50万亩，增粮1亿斤。全面落实各项支农惠农政策，大力发展设施农业、绿色农业，推进农业综合开发，加快建设一批规模大、特色强、集约化程度高的种植、养殖基地。支持发展家庭农场和专业大户，培育壮大农业产业化龙头企业，力争新增省级龙头企业5家以上。大力推进农田水利基本建设，新挖改造坑塘500个，新增节水灌溉面积30万亩。

不断增强科技支撑能力。大力实施创新驱动发展战略，充分发挥科技对产业结构调整的支撑引领作用。突出抓好创新能力建设，鼓励和引导企业加大研发投入，建立与高等院校、科研院所协同创新模式，推动产学研紧密联合，力争科技型中小企业达到2500家，高新技术企业109家。加强关键技术研发应用，提高科技成果转化率。推进招才引智工程，引进一批科技领军人才和创新创业团队。

（四）积极稳妥推进新型城镇化。坚持以人的城镇化为核心，以县域城镇化为重点，提升城市品质，改善农村面貌，推动城乡一体融合发展。

优化城镇发展布局。科学编制和完善城镇规划体系，促进城乡总体规划、产业发展规划、土地利用规划“三规合一”，实现城乡规划全覆盖、要素全统筹、建设一盘棋。坚持中心城区组团式发展方向，构建以主城区为核心，沧县、青县同步跟进、协调发展新格局。突出特色、完善功能，推动县级市和基础好、实力强的县城加快向中等城市迈进，培育和形成一批宜居宜业的高标准小城市。

加快中心城区建设。以创建国家园林城市和迎省十四运为抓手，着眼东西部城区均衡发展，重点围绕西部城区、东部新城、火车站周边、运河沿岸“四个片区”，推进实施一批重点城建工程。道路畅通工程：新建续建改造市政道路27条，打通育红路、铁西大街等6条“断头路”。依托黄河路建设城市快车道，解决市区东西向交通瓶颈问题。园林绿化工程：继续实施绿色行动三年计划，新建续建动物园、长芦、兴业等6个公园、10个游园，抓好26条道路绿化工程，新增绿化面积1053万平方米。加快万荣、五针松等城市森林公园建设，构筑城郊一体的防护林体系。环境提升工程：加快推进运河景观综合改造，打造高品质绿色生态走廊。加强城市管理，实施解放路、迎宾大道等5条道路环境综合整治工程，抓好北京路、上海路等13条道路景观亮化，全面提升市容环境卫生、道路交通管理水平。公用设施工程：加快城市排水、集中供热、运西污水处理厂扩建等工程建设，新建供水管线30公里，铺设燃气管线6公里、供热管线15公里，改造建设换热站39座。便民设施工程：新建生活垃圾收集、运转站24座，整治便民疏导街道20条，改造小街小巷40条，设立社区果蔬销售点100个，为广大市民创造更加便利舒适的生活环境。

打好县城建设攻坚战。以扩容提质为目标，合理调整县城空间结构，做大县城规模，满足产业布局和人口发展需要。集中优质资源，建成一批高水平的公共服务和大型商贸设施，打造一批标志性建筑和特色空间节点。大力开展环境容貌整治提升行动，提高县城规范化管理水平。全面放开县级市、县城和建制镇的落户限制，健全农业转移人口户籍管理制度，有序推进转移人口市民化，力争县域城镇化率提高1.5个百分点。

统筹城乡一体化发展。突出重点，分步实施，优先推进城市周边区域一体化建设，逐步向交通便利、基础条件好、人口相对集中的城镇覆盖。加快基础设施和公共服务向农村延伸，促进路电气讯等设施城乡联网、共建共享。深入推进农村面貌改造提升行动，打造一批精品特色村，持续改善农村生产生活条件。

（五）下大力改善生态环境。坚持经济发展与生态建设同步推进，进一步加大生态环境保护和污染治理力度，让沧州的天更蓝、地更绿、水更清，全市人民都能“深呼吸”。

加强环境污染治理。严格落实《大气污染防治行动计划实施方案》，加快淘汰燃煤小锅炉，加大小化工、小炼厂等重污染企业治理力度，抓好煤场、料场和建筑施工规范管理，全面禁止秸秆焚烧，完成淘汰“黄标车”3.6万辆和全部518辆燃油公交车更换任务。加强重点流域、饮用水源地和地下水污染防治，切实改善水环境质量。

扎实推进节能减排。严格落实节能减排目标管理责任制，实施节能技改项目100项，淘汰落后生产线30条。严把审批关口，坚决杜绝“两高一低”项目上马。加快污水处理厂、垃圾处理场建设，确保正常运营，发挥效益。大力推进建筑节能，创建绿色低碳城市。加快示范园区、示范企业建设，大力发展循环经济、清洁生产。

加大环境执法力度。积极构建政府主导、环保监管、部门协同、企业落实、社会参与的生态环境治理机制，强力开展“利剑斩污”行动，坚决依法打击各类环境违法行为，形成依法治理环境的强大震慑力。

（六）扎扎实实推进各项改革。坚持“正确、准确、有序、协调”方针，按照中央和省决策部署，积极推进重点领域改革。

深化激活市场主体改革。进一步简政放权，衔接落实好国务院和省政府取消、下放审批事项，除要求市级保留的事项外，全部下放到县，选择2个县（市）试点下放到乡。贯彻落实《行政审批制度改革实施意见》，精简审批事项，简化办事手续，优化工作流程。选择市本级20个部门、40项服务作为试点，推动政府向社会购买服务。深入推进商事登记制度改革，巩固扩大《改革企业登记制度实施办法》成果，变“先证后照”为“先照后证”，力争新增各类市场主体2.4万户。

深入推进国有、集体企业改革。加快国有、集体企业公司制改造和企业集团化步伐，建立现代企业制度，提高国有资产的运营效益和增值能力。大力发展混合所有制经济，鼓励民间资本参与国有企业改制重组，参股国有资本投资项目。

积极推进财政金融体制改革。改进财政预算管理制度，推进全口径预算编制改革，进一步提升政府财力统筹水平。强化地方政府债务管控，有效防范债务风险。大力发展金融机构和新兴金融业态，提升金融服务实体经济能力。整合运作各类资源，做大做强市、县两级投融资平台。推进企业上市融资，力争各县（市）新增1家上市企业。

稳步推进农村综合改革。积极推进土地承包经营权流转，推动适度规模经营。支持发展集体经济组织，壮大集体经济实力。加快推进农村土地分类确权登记颁证工作，探索建立农村产权交易市场，推动农村产权公开、公正、规范交易流转。

加快推进社会事业改革。统筹城乡教育资源均衡配置，完善家庭经济困难学生资助体系，大力促进教育公平。深入推进医药卫生体制改革，健全全民医保体系，建立城乡居民大病保险制度和重特大疾病救助机制。落实好中央和省各项社会保障改革措施，建立完善更加公平可持续的社会保障制度。

（七）着力提升对外开放水平。坚持以大开放促进大发展，进一步加大宣传推介和招商引资力度，加快构筑全方位、多层次、宽领域的对外开放新格局。

全面对接京津。紧紧抓住京津冀协同发展和北京亟需寻找新的疏解扩散空间重大机遇，充分发挥我市沿海临港、土地资源丰富、劳动力成本低等综合优势，积极开展产业、金融、教育、医疗等全方位对接，重点围绕京津大型央企、高科技园区组织实施系列主题招商活动，以优惠的政策和良好的环境，吸引一批先进制造业和高新技术产业项目，打造京津产业转移、科技创新基地，实现借势借力发展。

推进战略合作。瞄准港台、欧美等国家和地区，紧盯世界500强、国内500强，特别是石油化工、管道装备等行业龙头50强企业，动态掌握企业发展规划和投资动向，以资源引项目，以品牌换技术，力争在战略合作、资产重组上实现更大突破。

创新招商机制。按照“走出去、引进来”相结合的原则，精心组织各类大型经贸洽谈活动，开展好领导招商、产业链招商和常态化驻点招商，增强招商引资针对性和实效性。建立健全招商项目跟踪推进机制，实行专人负责、全程服务、一包到底，保障项目成功落地、顺利实施。

扩大外贸出口。强化外贸企业帮扶，培育特色品牌，重点抓好石油钻采设备、裘皮服装等出口基地建设。力争新增进出口超千万美元企业10家，进出口贸易总额达到27亿美元。

（八）更加注重保障和改善民生。坚持把人民群众的期待作为政府工作努力方向，先行保障民生投入、先行安排民生项目、先行解决民生问题，让发展成果更多更公平惠及全市人民。

推动实现更高质量就业。实施更加积极的就业扶持政策，多渠道开发就业岗位，促进以高校毕业生为重点的青年就业和农村转移劳动力、城镇困难人员、退役军人就业。健全城乡均等公共就业创业服务体系，鼓励支持全民创业。新增城镇就业5.68万人，失业登记率控制在4.5%以内。

稳步增加城乡居民收入。在确保经济平稳较快发展的同时，逐步提高机关事业单位工资和津贴补贴。大力发展非农产业，加强农民技能培训，鼓励农民就地转移，增加农民工资性收入。

协调发展各项社会事业。坚持教育优先，推进各级各类教育内涵发展。整合职教资源，提高高等教育办学层次和水平。探索建立市、县医疗机构集团化管理模式，促进医疗资源共享。积极发展医疗健康产业，打造区域医疗服务中心。支持发展中医药事业，提升基层中医药服务能力。大力发展文化产业，繁荣文化创作，努力打造一批艺术精品。积极开展全民健身活动，全力做好省十四运筹备组织工作，加快建设文化强市、体育名市。

切实维护社会安全稳定。严格落实安全生产责任制，突出抓好危险化学品、道路交通、人员密集场所等重点行业、重点区域监管，坚决遏制重特大安全事故发生。深入开展“食药安全、诚信沧州”行动，保障人民群众饮食用药安全。创新社会治理，畅通群众诉求表达渠道，及时化解矛盾纠纷。加强平安沧州建设，依法严厉打击各类违法犯罪活动，确保社会和谐稳定。

今年将重点实施一批民生建设工程。社会保障工程：扩大“五险合一”试点范围，巩固完善新农合制度。提高退休人员基本养老金和城乡居民社会养老保险水平。城乡居民医疗补助标准人均提高到320元，城乡低保标准分别达到5400元和2500元。保障性安居工程：建设棚户区改造和保障性住房1.5万套、竣工2.3万套，投入使用2万套。养老“幸福工程”：加强光荣院、敬老院、福利院建设，扶持发展社会办养老服务机构；继续实施农村社会养老“幸福工程”，完成200个建制村建设任务。教育提升工程：推进市一中迁建二期、职业技术学院及工贸学校二期等建设工程，创建市级示范性幼儿园50所、标准化中小学50所，支持河北工专申报本科院校，完成沧州师院本科教学评估验收。医疗设施建设工程：加快远程医疗服务平台建设，续建市人民医院医专院区，启动中心医院西院区二期、中西医结合医院和妇幼保健院综合楼工程。扶贫开发工程：确保8万贫困人口实现稳定脱贫。饮水安全工程：新建扩建大型水厂8座、分水厂14座，解决60万人的饮水安全；完成南水北调中线引水工程，让沧州人民喝上长江水。文体惠民工程：加强公共文化体育设施建设，完成260个村体育健身设施更新维修任务，市、县大型公共文化设施全部向社会免费开放。便民服务工程：开通“961890”群众服务热线，完善企业“110”服务中心运行管理机制，搭建更加便捷、高效的公共服务平台。平安创建工程：市区布设视频监控点860处、智能高清卡口168处，各县（市）城区分别完成200处以上视频监控建设任务，实现视频监控全覆盖。继续推进“一村一警三支队伍”警务机制，完善城乡社会治安防控体系。

加强国防教育和后备力量建设，巩固增进军政军民团结。认真做好第三次经济普查，支持工会、共青团、妇联等群众组织发挥桥梁纽带作用，统筹推进民族宗教、外事侨务、人口计生、妇女儿童、防震减灾，以及档案、气象、人防、老龄、残疾人等各项工作。

四、加强政府自身建设

建设沿海强市、和谐沧州，实现全市人民的美好愿景，政府责任重大。每一名政府工作人员都要时刻铭记全心全意为人民服务的宗旨，进一步强化发展意识、责任意识、公仆意识，着力建设为民、务实、清廉、高效政府。

坚持依法行政，不断提高科学决策的能力和水平。强化依法行政理念，严格按照法定权限和法定程序行使权力、履行职责，对违法行政行为予以严肃追责。坚持科学决策、民主决策，广泛听取人大代表、政协委员及社会各阶层、各界人士的意见和建议，确保政府决策得民心、顺民意，符合经济社会发展规律。

坚持对标先进，不断提高推动发展的能力和水平。继续开展解放思想、对标赶超活动，深入学习先进地区经验做法，认真查找在思想观念、体制机制、发展环境、社会管理等方面的差距，制定实施激发市场活力、推动经济社会发展的新举措、新办法，以对标挖潜力，以对标增动力，以对标促赶超。

坚持改进作风，不断提高求真务实的能力和水平。扎实开展党的群众路线教育实践活动，坚决抵制和克服“四风”，切实把思想和精力集中到爱岗敬业、干事创业上来。坚持求真务实，真抓实干，以踏石留印、抓铁有痕和钉钉子的精神，推动工作落实，在沧州大地唱响“实干兴沧”主旋律。

坚持倡廉治奢，不断提高廉洁从政的能力和水平。厉行勤俭节约，反对铺张浪费，严控“三公”经费支出，大幅降低行政成本。深入推进政府系统惩治和预防腐败体系建设，严格落实党风廉政建设责任制，教育引导各级干部进一步增强廉洁自律意识，坚持原则，慎用公权，守住底线，不谋私利，做到干部清正、政府清廉、政治清明。

各位代表，时代赋予我们的使命，光荣而神圣，人民寄予的希望，殷切而厚重。今天的沧州，站在了新的历史起点，进入了转型发展的关键时期，让我们在中共沧州市委的正确领导下，团结一心，抢抓机遇，开拓进取，扎实工作，为加快建设河北沿海地区率先发展增长极，创造更加幸福美好的明天而努力奋斗！
